# Supplementary material for: Ultrasensitive nano-optomechanical force sensor operated at dilution temperatures
Source: Nat Commun. 2021 Jul 5;12:4124. doi: 10.1038/s41467-021-24318-y (PMC8257768; doi:10.1038/s41467-021-24318-y)
Supplement: Supplementary file 1 — Supplementary Information [file 41467_2021_24318_MOESM1_ESM.pdf]

Supplementary Information - Ultrasensitive nano-optomechanical force sensor operated at dilution temperatures

Francesco Fogliano, Benjamin Besga, Antoine Reigue, Laure Mercier de Lépinay, Philip Heringlake, Clement Gouriou, Eric Eyraud, Wolfgang Wernsdorfer, Benjamin Pigeau, and Olivier Arcizet*

SUPPLEMENTARY NOTE 1 - SPECTRAL CONVENTIONS

We employ the following relations for spectral conventions, noise spectral densities and Fourier components:

$$A[\Omega] \equiv \int_{-\infty}^{\infty} dt A(t) e^{+i\Omega t} \quad \text{and} \quad A(t) \equiv \int_{-\infty}^{\infty} \frac{d\Omega}{2\pi} A[\Omega] e^{-i\Omega t} \tag{1}$$

so that the noise spectral density of the random variable A, defined by :

$$2\pi\delta(\Omega+\Omega')S_A[\Omega] = \langle A[\Omega]A[\Omega']\rangle \tag{2}$$

where the average is considered over many realizations of the random process, is related to the temporal autocorrelation function:

$$S_A[\Omega] \equiv \int_{-\infty}^{\infty} d\tau C_A(\tau) e^{i\Omega\tau} \tag{3}$$

With those conventions, the spectral density of the Langevin force noise reads, according to the fluctuation dissipation theorem:

$$S_F[\Omega] = \frac{2k_BT}{\Omega} Im \frac{1}{\chi[\Omega]} = 2M_{\mathrm{eff}}\Gamma k_B T \tag{4}$$

where the nanowire mechanical susceptibility is given by $\chi[\Omega]^{-1} = M_{\mathrm{eff}}(\Omega_{\mathrm{m}}^2 - \Omega^2 - i\Omega\Gamma)$. This is called the double sided convention. This definition thus leads to a force sensitivity (in $\mathrm{N/Hz}^{1/2}$) $\sqrt{2}$ times smaller than the single sided convention. This has to be taken into account when comparing the different systems.

SUPPLEMENTARY NOTE 2 - OPTICAL SCHEME

A more complete optical scheme of the experiment is shown in Supplementary Figure 1. A 2 mW laser, 632.8 nm is attenuated down to 100 fW using a combination of wave-plates, polarising beam splitters and polarisers, followed by a single path acousto-optic modulator (MT200-VIS-0.5 from AA). The latter permits to regulate the injected optical power and to adjust it electronically. A 90:10 or a 99:01 non-polarizing beam splitter cube serves as a last

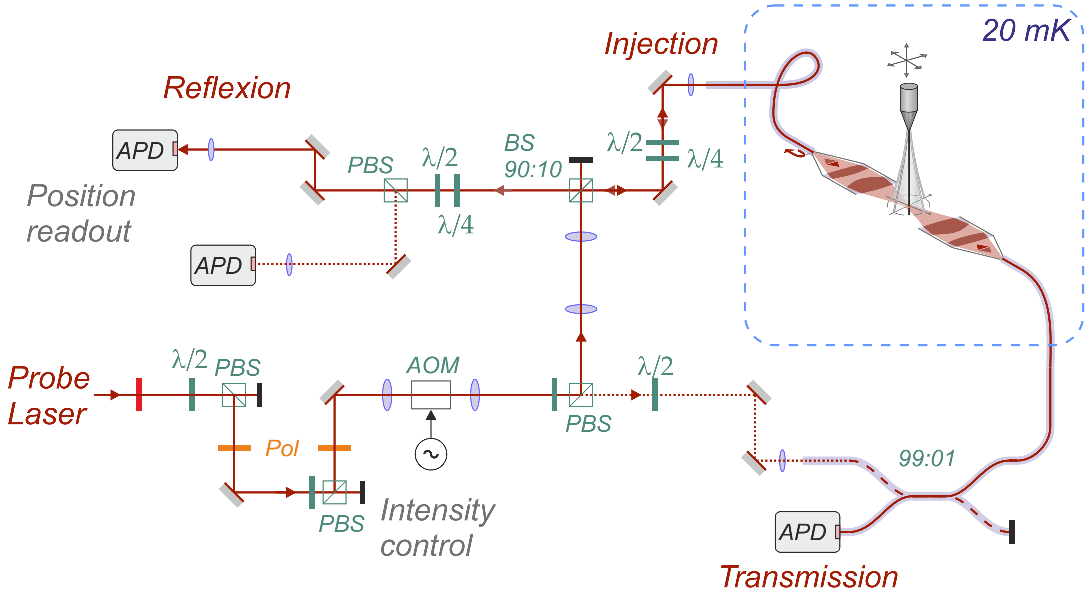

Supplementary Figure 1. **Schematics of the optical scheme** BS: beam splitter, PBS: polarizing beam splitter, APD avalanche photodiode, AOM :acousto-optic modulator.

attenuator and allows to collect 90% of the light reflected from the cryostat. The laser is filtered by a notch filter to eliminate the parasitic visible photons that can otherwise be channeled to the experiment and to the detectors. A set of wave-plates allows to adjust the injected polarisation with respect to the nanowire axes, while compensating for the ellipticity appearing in the propagation. A second set of wave-plates can be used to adjust the optical contrast in the readout channels, using the fact that the reflection on the nanowire induces some birefringence (see below). Two single photon counters (Perkin Elmer ARQH16) can be employed in reflection, or in the transmission channel. If necessary the beam splitter can be replaced by a fiber beam splitter, as shown in the transmission channel. The electrical pulses produced by the APDs are sent into pulse splitters that provide identical signals to several detection apparatus, such as photon counters, photon correlators, spectrum or network analyzers.

The fibers employed are broadband visible fibers (SM600) featuring APC terminations in order to suppress unwanted stationary waves inside the optical path, which would compromise the readout interference stability. Fiber connectors are also avoided, we do not use vacuum feed-through to enter the cryostat and instead splice the fibers when needed. The fiber objectives are made of highly conductive copper. The fiber is maintained in a ceramic ferule, and two aspheric lenses, with focal length of 16 and 3 mm are employed, anti-reflection coated. The concentricity of the system is essential to provide a decent optical interference pattern, in particular, a specific care is taken in the fiber cleaving angle, which should be as close as possible from 90 degrees, while the mechanical tolerances are extremely tight. The centering of the fiber core into the fibers is essential to obtain a correct wavefront pattern. For the final adjustment of the system, and in particular to adjust the fiber insertion depth into the ferule support, we realize nanowire-based scanning probe images of the wavefronts, as shown in the article, Supplementary Figure 1f. In this optimization phase, we rotate the fiber and adjust its position within the ferule, and modify the cleaving angle if the wavefront structure is not perfectly symmetric. The quality of the interference pattern is preserved at low temperatures and no visible changes are observed. The fiber objectives have supported tens of temperature cool downs, without showing any apparent degradation. They present a relatively large chromatism, with an axial separation of approx. 15 μm between the waists produced using 532 and 633 nm laser wavelengths. This chromatism is finally found to be an advantage for pump-probe experiments realized using both fiber objectives, since it limits the transmission of unwanted light. The fiber to fiber transmission can be optimized above 50 percent, despite only using translation degrees of freedom.

SUPPLEMENTARY NOTE 3 - THE NANOWIRES

Silicon carbide nanowires: properties and preparation

Silicon carbide is a large band gap material. It allows operating with focussed visible light without destroying the nanowires: they can sustain tens of mW at room pressure, and up to a few mW at low pressure without degrading. Here we used 3C CVD grown crystalline nanowires, which present very few defects along their length, which can reach hundreds of microns, and diameters spanning from 100 to 400 nm typically. We operate with a powder of nanowires, select the nanowires and glue them at the extremity of sharp tungsten tips, which have been electrochemically etched in a KOH solution. The nanowires of interest are selected under an optical microscope, exploiting their diameter-dependent color observed in reflection under polarized white light illumination. A numerical simulation making use of the Mie scattering properties of the nanowires allows a precise determination of their diameter without using SEM imaging which can lead to sample contamination. Depending on their diameter, the Mie resonances can lead to an appreciable increase of the scattering cross section, see [1], which permits obtaining large optical contrast in the visible.

Once mounted on their metallic support, the nanowires are cleaned from unwanted dust or from other attached nanowires using the surface tension of a small water drop inside which the nanowire is slowly immersed, allowing to efficiently clean the nanowire. The nanowire length can be subsequently adjusted using laser cutting at high power (100 mW). The nanowires are then mounted in a large conductivity copper support frame, where it is set using an hydraulic press. The nanowire can be baked at approx. 600 degrees in secondary vacuum, which helps improving their mechanical quality factor at room temperature, through an hardening of the glue. However we found that this process does not have a meaningful impact on their low temperature quality factors, and can be responsible for metallic deposition leading to an increased optical absorption.

The nanowires present an oxyde amorphous crust, as revealed in TEM imaging [2] of a few nm in thickness, which is the dominant cause of mechanical dissipation at low temperatures and could have an appreciable impact on the nanowire conductance at low temperatures.

Using silicon carbide also allows to operate with a rather light and stiff material, so that the mechanical frequencies

remain reasonably large, even for the ultra-long aspect ratios employed in this work. Operating at frequencies smaller than 500 Hz would be problematic due to the vibration noise level, which will not be efficiently filtered out by the suspension apparatus. At several steps in the fabrication process, it can be delicate to operate with ultra-soft nanowires: under the influence of the air flow occurring during by their manipulation they can bend and irremediably stick to their support. This renders difficult to operate in the upper left area of the following abacus plot, due to their extremely low stiffness. With a careful handling of the nanowires, force sensitivities in the 10 zN/Hz$^{0.5}$ range should be accessible with similar nanowires.
Removing the oxyde crust should in principle help increasing the probe mechanical quality factors, however this remain an ongoing work.

Nanowire samples

NW	L(μm)	d(nm)	$\Omega_m/2\pi$(kHz)	Q at20 mK	M_{eff}(pg)	dF_{min}(zN/Hz$^{1/2}$)	T_{eff}^{min}
NWA	330	300	2.95	75 000	16	60	32
NWB	216	175	5.84	95 000	4	51	62
NWC	245	240 − 120 (conical)	11.6	96 000	1.6	40	47

Supplementary Table I. **Nanowires discussed in this work** The last columns represent the minimum noise temperature and the minimum force noise measured.

Size dependent properties

The nanowire dimensions - length L and diameter d - have a large impact on the mechanical and optical properties of the force probes. Depending on the type of experiment envisioned, one has to employ the adequate force sensor. Here follows a brief review of their main characteristics. The source of nanowire we use have a diameter spanning from 80 to 500 nm typically, for lengths up to a mm. However it can become very delicate to operate with too soft nanowires, featuring stiffness of a few 100 nN/m, in particular because air flows occurring during the preparation phase make the nanowire bend and stick to its support.

Frequency

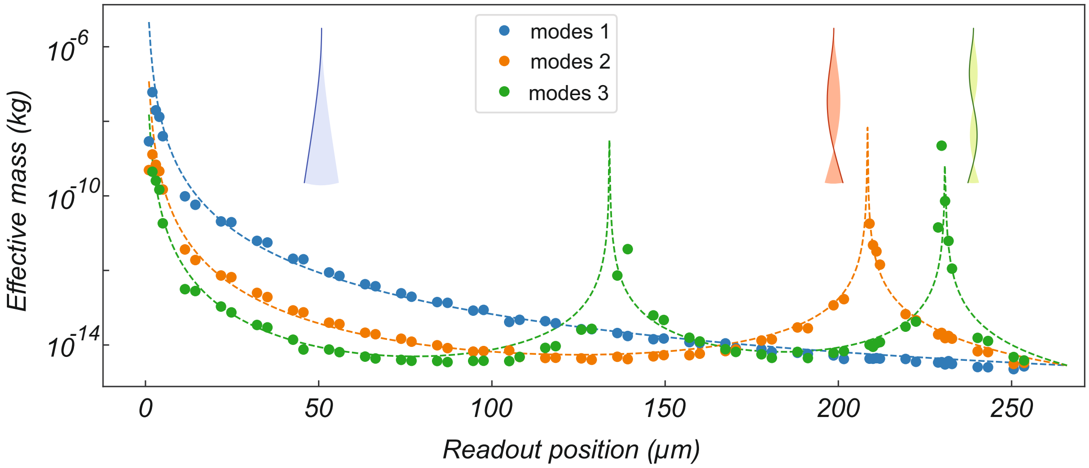

Supplementary Figure 2. **Longitudinal dependence of the effective mass.** Dependence of the transverse mode profiles with the readout position along the nanowire, expressed in term of effective mass, for the first 3 longitudinal eigenmodes of a $\approx 250\,\mu$m nanowire of 130 nm diameter. When the laser is positioned within the last 20 percents of the nanowire length, the effective mass is increased by less than 2 compared to its minimal effective mass, measured at its extremity.

The fundamental frequency $\Omega_\mathrm{m}/2\pi$ of a singly clamped nanowire is given by the Bernouilli formalism:

$$\Omega_\mathrm{m}/2\pi = 0.14\sqrt{\frac{E_\mathrm{SiC}}{\rho_\mathrm{SiC}}}\frac{d}{L^2} \propto d/L^2 \tag{5}$$

where we use a density of $\rho_\mathrm{SiC} = 3210\,\mathrm{kg/m^3}$ and a Young modulus of $E_\mathrm{SiC} = 380\,\mathrm{GPa}$ for our batch our nanowires. It is shown in red in Supplementary Figure 3 and the frequencies achieved range from few hundreds of Hz to several hundreds of kHz.

Effective mass

When the probe laser is positioned at the extremity of the nanowire, the effective mass M_eff amounts a quarter of the total mass of the nanowire:

$$M_\mathrm{eff} = 0.25\rho_\mathrm{SiC}L\left(\frac{d}{2}\right)^2 \propto L.d^2 \tag{6}$$

It is shown in green in Supplementary Figure 3, and the masses employed range from 0.1 pg to 100 pg.

Force sensitivity

Using the double sided spectral convention, the limit imposed on the force sensitivity by the Langevin force noise $\delta F_\mathrm{min}[\Omega]$ (in $\mathrm{N/Hz^{1/2}}$) is given by:

$$\delta F_\mathrm{min} = \sqrt{2M_\mathrm{eff}\Gamma_\mathrm{m}k_BT} \propto d^{3/2}/L^{1/2} \tag{7}$$

where we have assumed a fixed quality factor (Q=100000) so that $\Gamma_\mathrm{m} \propto \Omega_\mathrm{m}$. The diameter has a dominant impact on the force sensing capacity of the nanowire probes. In practice, it can be very difficult to prepare ultrasoft nanowires (stiffness below $1\,\mu$N), due to their too large bending capacity, which makes them easily stick to their support in presence of air flows.

Thermal noise amplitude

The resonance of the thermal noise peaks at a value of

$$S_{\delta r_\beta}[\Omega_\mathrm{m}] = \frac{2k_bTQ}{M_\mathrm{eff}\Omega_\mathrm{m}^3} \propto L^5/d^5 \tag{8}$$

SUPPLEMENTARY NOTE 4 - OPTICAL PROPERTIES AND REFLECTION EFFICIENCY

Silicon carbide is a large band gap material, with a gap ranging from below. 400 to 515 nm for its most common allotropic phases (4H/6H and 3C phases respectively) and presents a rather large refractive index, around 2.7 in the visible. For the diameters we employ, in the 100-400 nm range, this allows the nanowire to sustain internal optical resonances for optical wavelengths in the visible. Those internal modes are called Mie resonances, and can be computed analytically [1] in the case of an infinite cylinder illuminated by a monochromatic radiation. The original theory assimilates the incoming light field to a plane wave, and permits to compute the fields inside and outside of the nanowire, for any polarization of the incoming light field, and any orientation of its Poynting vector. It is then possible to describe the scattering properties of the nanowire to any incoming light field by expanding it on the plane wave basis.

We have seen in the above section that minimizing the nanowire diameter is the key ingredient to increase the nanowire force sensitivity. It is thus important to precisely determine the diameters of the nanowires we employ. This can be done in a SEM but for very long nanowires it s very difficult to image the vibrating extremity of the nanowires, due to vibrations and actuation by the readout electron beam, and can furthermore create some decontamination of the nanowire, potentially increasing the optical absorption rate. We then prefer to implement a pure optical method, realized by simply imaging the nanowires under a white-light microscope objective, and determining their color for both polarizations of the incoming light source. This requires to take carefully into account the chromatic rendering of

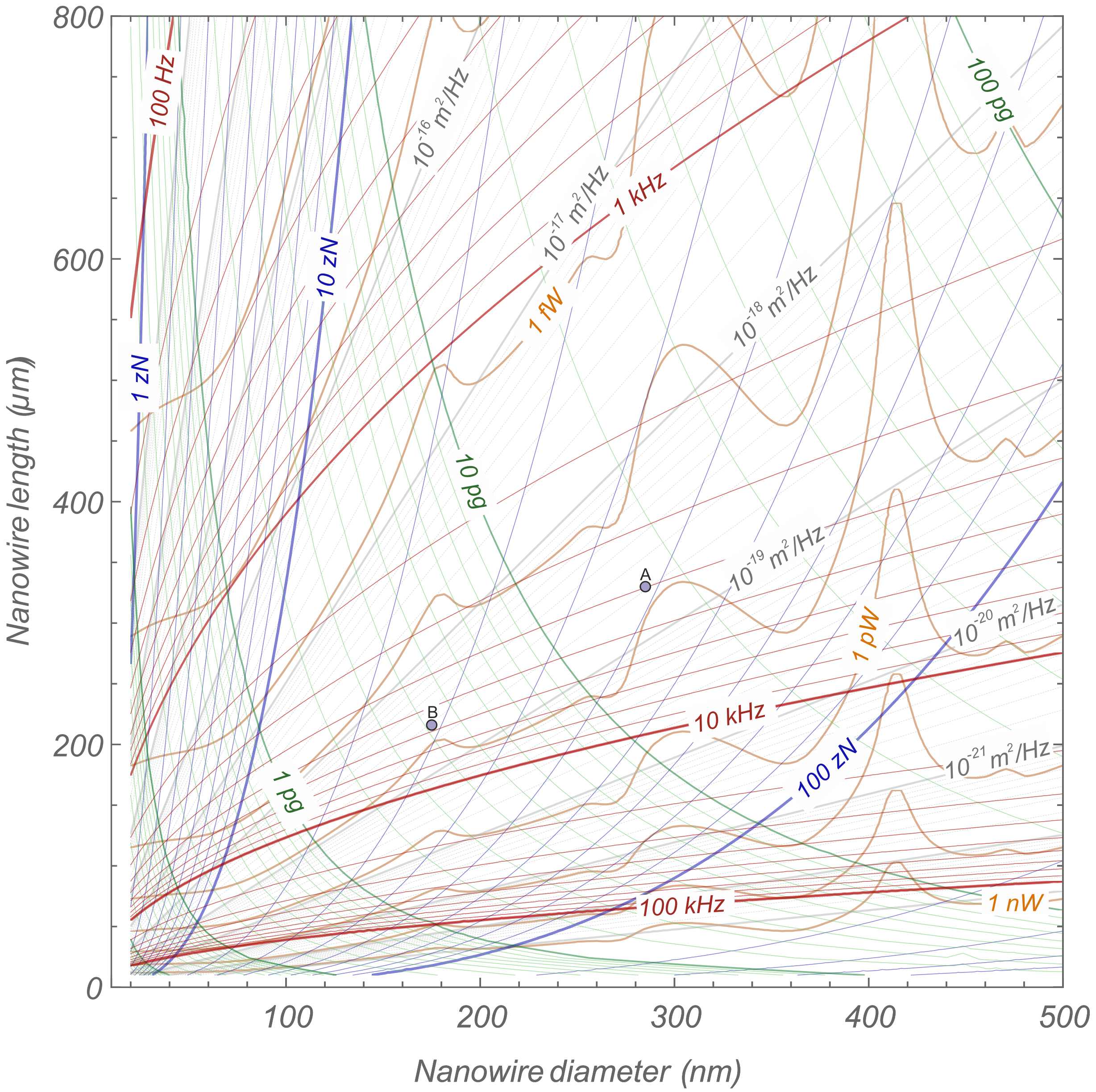

Supplementary Figure 3. **Abaques.** Dependence on the nanowire geometry - assumed to be perfectly cylindrical here - of the nanowire fundamental resonance frequency (red), its effective mass (grenn), its force sensitivity $\delta F_{\rm min}$ (blue), and resonant thermal noise amplitude $S^{\rm th}_{\delta r_\beta}[\Omega_{\rm m}]$ at 20 mK (gray) assuming a constant quality factor all over the map (Q=100 000). The orange lines are the injected power required to detect their thermal noise peak with a signal to shot noise of 1. The positions of the cylindrical nanowires A and B employed in the article are highlighted (nanowire C is conical, contrary to the cylindrical assumption employed to establish the abacus.)

the CCD camera and of the screen employed, see ref. [3]. This method is extremely efficient to estimate the nanowire diameters with a precision around ±5 nm and also serves to detect any conical shape.

The optical contrast in reflection is numerically simulated using the Mie scattering formalism, combined with a description of the focused laser beam which permits to include non paraxial polarization effects (even if in the following, we will assume the nanowire is localized on the optical axis). We use a reference power reflected on the fiber output corresponding to $r^2_{\rm fiber} = 4\%$ of the incoming light. To compute the reflection coefficient of the focussed laser beam, $r^0_{\rm nw}$, we first expand the incoming Gaussian field on the plane wave spectrum. The polarization is introduced by assigning a different polarization vector for each contribution of the expansion [4]. We consider two cases, the parallel and perpendicular ones where the polarization vectors associated to the incident wave vector $\mathbf{k}_j$ of the expansion is

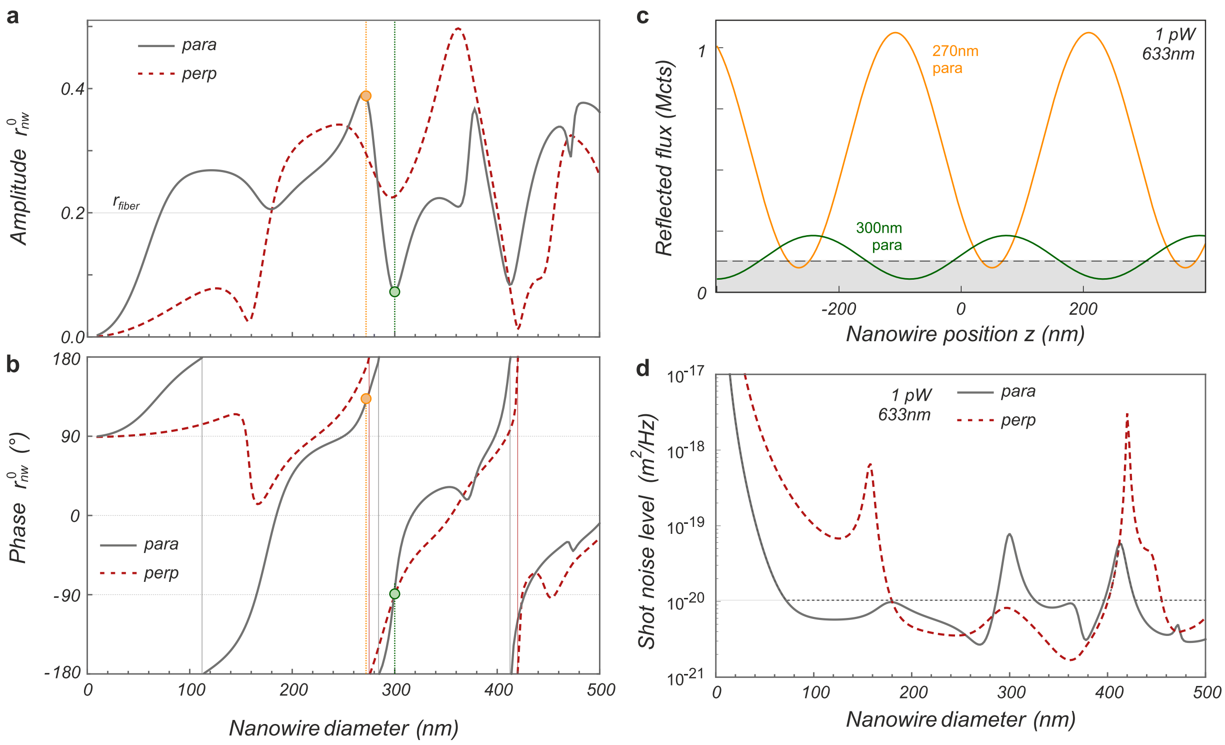

Supplementary Figure 4. **Optical contrast and shot noise level.a, b** Dependence of the amplitude and phase of the effective reflection coefficient $r^0_{\rm nw}$ with the nanowire diameter for laser light polarizations parallel and perpendicular to the nanowire axis (optical wavelength of 633 nm, numerical aperture of 0.5, $w_0 = 350\,$nm). The fiber reflection coefficient $r_{\rm fiber}$ is also indicated as a dotted line. The large variations seen in the amplitude and phase of $r_{\rm nw}$ are due to internal Mie resonances, which renders the reflected interference pattern shown in panel **c** strongly dependent on the nanowire diameter and light polarization. **c** Simulations of interference patterns $\phi_{\rm R}$ measured on the reflection detector, for 2 different diameters for the parallel polarization, in the two situations where the amplitude of $r_{\rm nw}$ is larger or smaller than $r_{\rm fiber}$. The laser waist is located at $z = 0$, and the injected power is 1 pW. The amount of light reflected in absence of nanowire is highlighted in gray. **d** Dependence of the shot noise level on the nanowire diameter for an injected power of 1 pW. The dotted line corresponds to the maximum of sensitivity obtained with $r_{\rm fiber} = 0.2$, while the solid and dashed lines are the best sensitivities that can be obtained for the 2 polarizations after optimization of the interferometer (input fiber reflectivity). When the reflection coefficient is found to be very anisotropic, several techniques based on polarization manipulations can be employed to maximize the SNR of the measurement. To build up the map shown in fig.3 of the minimum injected power required to detect the thermal noise peak, we have employed the maximum of sensitivity between both laser polarizations.

respectively in or orthogonal to the incidence plane $(\mathbf{k}_j, \mathbf{e}_y)$, $\mathbf{e}_y$ being parallel to the nanowire axis. This approach permits to correctly describe the polarization state of focussed laser beams [4]. We then use the Mie formalism [1] to compute the scattered field for each plane wave appearing in the Gaussian beam expansion, the total scattered field being the sum of each contributions. Finally, we consider that the only part of the scattered field collected by the fibred optical set-up is its projection on the incoming Gaussian beam, making use of the orthogonality of the Hermite-Gaussian modes.

Those calculations permits to estimate the amplitude and phase of the complex reflection coefficient of the nanowire, $r^0_{\rm nw}$, which is evaluated for a 633 nm laser wavelength, and reported in Supplementary Figure 4ab for the two perpendicular laser polarizations. One can clearly see the presence of optical Mie resonances, which structure the reflection coefficient. We note that for small diameters, the nanowire recovers a dipole like optical response which is only polarisable for input light parallel to its axis. The reflection coefficients $r_{\rm nw}$ and $r_{\rm fiber}$ completely determine the efficiency of the optical readout. The corresponding interference pattern measured in reflection can be written as:

$$\Phi_R(z) = \Phi_0 \left| r_{\rm fiber} + r^0_{\rm nw} t^2_{\rm fiber} \exp\left(2ikz\right)\right|^2, \qquad (9)$$

it is shown in Supplementary Figure 4c in two different situations for an input power of 1 pW. In the following, we will use $t^2_{\rm fiber} = 1 - r^2_{\rm fiber}$ and $r_{\rm nw} \equiv r^0_{\rm nw} t^2_{\rm fiber}$, which represents the fraction of the light field reflected from the nanowire

which participates to the interference.

We now investigate the signal to noise ratio of the position readout to complement the abacus shown above and estimate the minimum optical power required to detect the nanowire thermal noise. To do so, we first introduce the specificities of continuous position measurements realized with avalanche photodiodes in the photon counting regime.

SUPPLEMENTARY NOTE 5 - POSITION SENSING WITH PHOTON COUNTING PHOTODIODES

Here we explain how the time-modulated photon flux recorded on an avalanche photodiode operating in the Geiger mode can be employed to quantify the oscillator noise spectral density. The input signal is made of a series of voltage pulses:

$$V_R(t) = \sum_i V_\pi(t - t_i) \tag{10}$$

where the pulses are defined by the height V_π and their duration T_π as (see Supplementary Figure 5):

$$V_\pi(t) = V_\pi \quad \text{if} \;\; 0 < t < T_\pi, \;\; 0 \;\; \text{otherwise.} \tag{11}$$

The probability $dp(t)$ to detect a photon between t and $t + \delta t$ depends on the position of the nanowire extremity $\mathbf{r}(t) = \mathbf{r_0} + \delta\mathbf{r}(t)$ in the interference map $\Phi_R(\mathbf{r})$:

$$dp(t) = dt\, \Phi_R(\mathbf{r_0} + \delta\mathbf{r}(t)) \simeq dt\Phi_R(\mathbf{r_0}) + dt \;\; \nabla\Phi_R|_{\mathbf{r_0}} \cdot \delta\mathbf{r}(t) \tag{12}$$

In practice, the signals are acquired over a duration T, their spectral density is computed for each segment and subsequently averaged. The autocorrelation function of the input signal recorded during a single acquisition (of duration T) is given by:

$$C_{V_R}(\tau) \equiv \frac{1}{T}\int_0^T dt V_R(t)V_R(t-\tau) = \sum_i \int_0^T \frac{dt}{T} V_\pi(t-t_i)V_\pi(t-t_i-\tau) + \sum_{i\neq j}\int_0^T \frac{dt}{T} V_\pi(t-t_i)V_\pi(t-t_j-\tau) \tag{13}$$

where we split the sum into equal and different photon arrival times and the sums run on all the photon collected during an acquisition T. The first integral does not depend on t_i and is associated to the autocorrelation function of the pulse shape:

$$C_\pi^T(\tau) \equiv \int_0^T \frac{dt}{T} V_\pi(t)V_\pi(t-\tau) \tag{14}$$

It can be computed (see Supplementary Figure 5):

$$C_\pi^T(\tau) = \frac{V_\pi^2 T_\pi}{T} \;\; (1 - |\tau|/T_\pi) \quad \text{if} \;\; |\tau| < T_\pi, \; 0 \;\; \text{otherwise.} \tag{15}$$

The first sum is simply proportional to the number of photons recorded during the acquisition, which amounts in average to $\Phi_R(\mathbf{r_0})T$.

Using a change of variables $t \to t - t_i$, the second integral is indeed equal to $C_\pi^T(\tau - t_i + t_j)$. The signal autocorrelation function can then be written:

$$C_{V_R}(\tau) = \sum_i C_\pi^T(\tau) + \sum_{i\neq j} C_\pi^T(\tau - t_i + t_j) \tag{16}$$

The noise spectral density of the signal is computed by the spectrum analyzer:

$$S_{V_R}[\Omega] \equiv \int d\tau C_{V_R}(\tau) e^{i\Omega\tau} \tag{17}$$

We introduce the Fourier transform of the pulse autocorrelation function:

$$C_\pi^T[\Omega] \equiv \int d\tau C_\pi^T(\tau) e^{i\Omega\tau} \tag{18}$$

which can be computed:

$$C_\pi^T[\Omega] = \frac{T_\pi^2 V_\pi^2}{T} \mathrm{sinc}^2(\Omega T_\pi/2) \tag{19}$$

where the sinus cardinal function produces a cutoff in frequency at the inverse of the pulse duration (approx 20 MHz typically).

Experimentally, we average over several acquisition sequences of duration T. This permits to average over many noise realizations (mechanical and optical noises), and in particular, to also average over many realizations of the pulses sequences for an" hypothetical" given mechanical trajectory. This means that the first term which is proportional to the number of photon detections during the acquisition time, and do not depend on mechanical trajectories, will then become: $\Phi_R(\mathbf{r_0})\ TC_\pi^T[\Omega]$. It represents the shot noise contribution and can thus be written:

$$S_{V_R}^{\mathrm{shot}}[\Omega] = \Phi_R(\mathbf{r_0})\ T_\pi^2 V_\pi^2 \mathrm{sinc}^2(\Omega T_\pi/2). \tag{20}$$

The average of the second spectral integral, can be estimated by using

$$\int d\tau \int_0^T dt_1 \int_0^T dt_2 e^{i\Omega\tau} C_\pi^T(\tau - t_1 + t_2)\langle \Phi_R(t_1)\Phi_R(t_2)\rangle \tag{21}$$

where we replaced the sum over different arrival times by an integral form, weighting the times of arrival by the mean probability to detect a photon at the corresponding time: $dp(t_1), dp(t_2)$ introduced above. The symbol $\langle ...\rangle$ stands for average over many realizations of the thermal noise. We can expand it at first order to make the dynamical contribution appear :

$$\Phi_R(t_i) = \Phi_R(\mathbf{r_0}) + \left| \boldsymbol{\nabla}\Phi_R|_{\mathbf{r_0}} \right| \delta r_\beta(t_i) \tag{22}$$

When computing the average product, the terms linear in δr_β will average to zero, while the first static term is not of dynamical interest and has implicitly already been taken into account in the identical time discussion above in case of a pure shot noise situation (no correlations between different arrival times). As such the second integral can be written:

$$S_{V_R}^{\mathrm{signal}}[\Omega] = \left| \boldsymbol{\nabla}\Phi_R|_{\mathbf{r_0}} \right|^2 \int d\tau \int_0^T dt_1 \int_0^T dt_2 e^{i\Omega\tau} C_\pi^T(\tau - t_1 + t_2)\langle \delta r_\beta(t_1)\delta r_\beta(t_2)\rangle \tag{23}$$

If we now employ a change in variable: $\tilde{\tau} = \tau - t_1 + t_2$, we obtain:

$$S_{V_R}^{\mathrm{signal}}[\Omega] = \left| \boldsymbol{\nabla}\Phi_R|_{\mathbf{r_0}} \right|^2 \int d\tilde{\tau} e^{i\Omega\tilde{\tau}} C_\pi^T(\tilde{\tau}) \int_0^T dt_1 \int_0^T dt_2 \langle \delta r_\beta(t_1)\delta r_\beta(t_2)\rangle e^{i\Omega(t_1 - t_2)} \tag{24}$$

The first integral is simply $C_\pi^T[\Omega]$ as defined above, while the second double integration can be expressed by using the autocorrelation function of the projected oscillator position: $C_{\delta r_\beta}(t_2 - t_1) = \langle \delta r_\beta(t_1)\delta r_\beta(t_2)\rangle$ whose Fourier transform

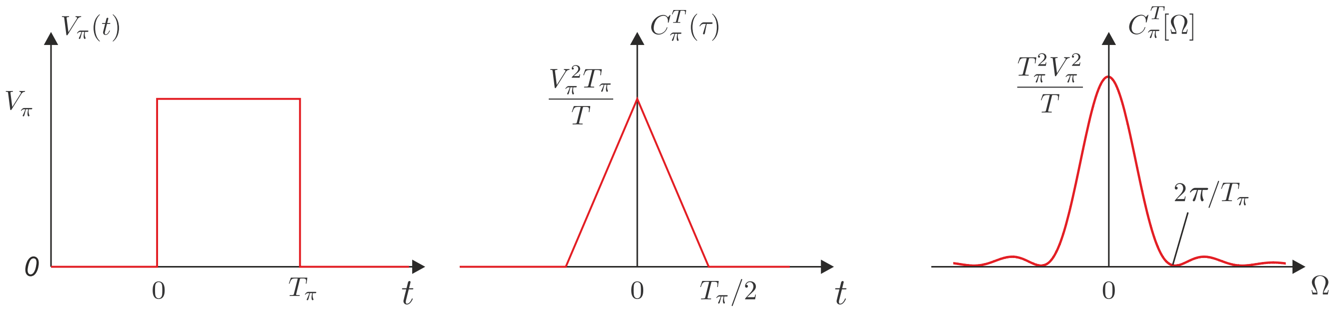

Supplementary Figure 5. Left: voltage pulse produced by the APD for a single photon detection event. Center: pulse autorcorrelation function. Right: Fourier transform of the autocorrelation function.

is the noise spectral density of the oscillator's position fluctuations:

$$S_{\delta r_\beta}[\Omega] \equiv \int d\tau C_{\delta r_\beta}(\tau) e^{i\Omega\tau}, \tag{25}$$

which is the quantity we are interested in recording experimentally. The last temporal integration simply gives an additional T contribution which cancels the one present in $C_\pi^T[\Omega]$. As such, the last integral can be expressed as:

$$S_{V_R}^{\mathrm{signal}}[\Omega] = \left|\boldsymbol{\nabla}\Phi_R|_{\mathbf{r_0}}\right|^2 T C_\pi^T[\Omega] S_{\delta r_\beta}[\Omega]. \tag{26}$$

so that the noise spectral density of the measured sequence of pulses reads:

$$S_{V_R}[\Omega] = T_\pi^2 V_\pi^2 \mathrm{sinc}^2(\Omega T_\pi/2)\left(\Phi_R(\mathbf{r_0}) + \left|\boldsymbol{\nabla}\Phi_R|_{\mathbf{r_0}}\right|^2 S_{\delta r_\beta}[\Omega].\right) \tag{27}$$

This formulation is rather explicit, the shot noise level is proportional to the received photon flux, while the signal part is transduced by the slope of the interference map. The overall signal is filtered for frequencies larger than $1/T_\pi$ (tens of MHz). In our experiments, we are only interested in the mechanical signals found at low frequency, so that the conversion between the voltage noise spectral density measured by the spectrum analyzer in the frequency representation ($S_\pi^f = 2\pi S_\pi[2\pi f]$) can be written as:

$$S_{\delta r_\beta}[\Omega] = \frac{S_{V_R}^f[\Omega]}{\eta^2 \left|\boldsymbol{\nabla}\Phi_R|_{\mathbf{r_0}}\right|^2}, \tag{28}$$

with

$$\eta = \sqrt{2\pi}\, T_\pi V_\pi. \tag{29}$$

This conversion factor between voltage and flux amounts to $\eta \approx 3.9 \times 10^{-8}$ V/Hz using $T_\pi = 15$ ns and a 3.3 V pulse magnitude produced by the photon counter, followed by a 10 dB attenuator ($V_\pi = 1.04$ V). We will see that this estimation provides a good order of magnitude for the η conversion factor.

In practice, there are many limitations such as the finite sampling rate of the spectrum analyzer (100 MS/s used), impedances mismatching or other sources of pulses distortions, so that it is necessary to independently calibrate the "volt to clics" η conversion factor. To do so, we generate an artificial optical noise using a AOM fed by a voltage trace mimicking the oscillator thermal noise, played by an arbitrary voltage generator. The optical outpout is then simultaneously readout on standard and APD photodiodes, using calibrated attenuators not to saturate the APD. The mean voltage and photon flux are recorded on the acquisition card, while the noise spectral density of the continuous and pulse voltage outputs are simultaneously recorded on a spectrum analyzer. After having compensated the power imbalance, it is possible to compared the noise spectral densities on each channel for identical input powers, and compute the η factor, which amounts to $\eta = 3.98 \times 10^{-8}$ V/Hz, in good agreement with the above estimation.

We also note that all the devices that record the vibrations of the nanowire, such as the spectrum analyzer, the network analyzer, the lock'in detection or the oscilloscope all present a 50 ohms input impedance, so that the same calibration factor η is used as soon as one wants to extract calibrated mechanical signals out of the avalanche photodiode photon fluxes. To prevent pulse reflections and distortions, we employed a "pulse splitter" unit which was developed to split the APD output into equivalent TTL outputs channels (x5), without being impacted by impedance management considerations.

SUPPLEMENTARY NOTE 6 - READOUT SENSITIVITY

From the above section, the minimal nanowire thermal noise level which can be detected above the shot noise level can be written as:

$$S_{\delta r_\beta}^{\text{shot}} = \frac{\Phi_R(\mathbf{r_0})}{\left|\boldsymbol{\nabla}\Phi_R|_{\mathbf{r_0}}\right|^2}, \tag{30}$$

so that one should generally try to maximize the measurement slope, while trying to operate at minimal mean optical flux. The maximum signal to background (SNB) is obtained by minimizing the above expression for a given input power. Since the nanowire can move along the 2 perpendicular orientations, it is necessary to also consider the direction of the measurement angle $\mathbf{e}_\beta$ as a parameter for the optimization if one wants to consider a particular eigenmode, knowing that it is always possible to turn the nanowire to align the mode of interest with the direction of the measurement vector.

If we restrict the nanowire motion to z axis, as explained above, we can describe the $\lambda/2$ periodic interference pattern as:

$$\Phi_R(z) = \Phi_0 \left|r_{\text{fiber}} + r_{\text{nw}} \exp\left(2ikz\right)\right|^2, \tag{31}$$

where $r_{\text{fiber}}, r_{\text{nw}}$ represent the complex reflection coefficients in amplitude from the fiber output and from the nanowire (including the fiber transmission coefficient, see above), $k = 2\pi/\lambda$ is the wave vector. $\Phi_0 r_{\text{fiber}}^2$ represents the reflected flux measured in absence of nanowire, which amounts to c.a. $r_{\text{fiber}}^2 = 4\%$ of the injected power. The effective reflection coefficient from the nanowire $r_{\text{nw}} e^{2ikz}$ can be evaluated for any position within the optical waist and any diameter of the nanowire, by computing the reflected EM field produced by the nanowire (while taking into account the Mie resonances of the nanowire), and projecting it back onto the illuminating light mode. From our numerical simulations, see Supplementary Figure 4, the light reflected from the nanowire $\Phi_0 r_{\text{nw}}^2$ can amount to up to 25 percents of the incoming light flux, i.e. up to 6 times the one reflected from the fiber output.

In the following, for simplicity we will assume that r_{nw} does not depend on the position z, which is true as long as one stays within the Rayleigh length of the beam, and take it as a real number, since any dephasing will at first order simply displace the interference pattern along the z axis. The shot noise limited background is then given by $S_{\delta r_\beta}^{\text{shot}} = \Phi_R(z)/\left(\partial_z \Phi_R(z)\right)^2$ while the SNR is proportional to the inverse expression and is explored in Supplementary Figure 6 for varying values of r_{nw}. For a small nanowire reflectivity compared to the fiber reflectivity ($r_{\text{nw}} << r_{\text{fiber}}$), the interference pattern is not very contrasted, and the position which minimizes the shot noise level is close to the side of the fringe position (found at $z = \lambda/8, 3\lambda/8$). For larger nanowire reflectivity, still verifying $r_{\text{nw}} < r_{\text{fiber}}$, the

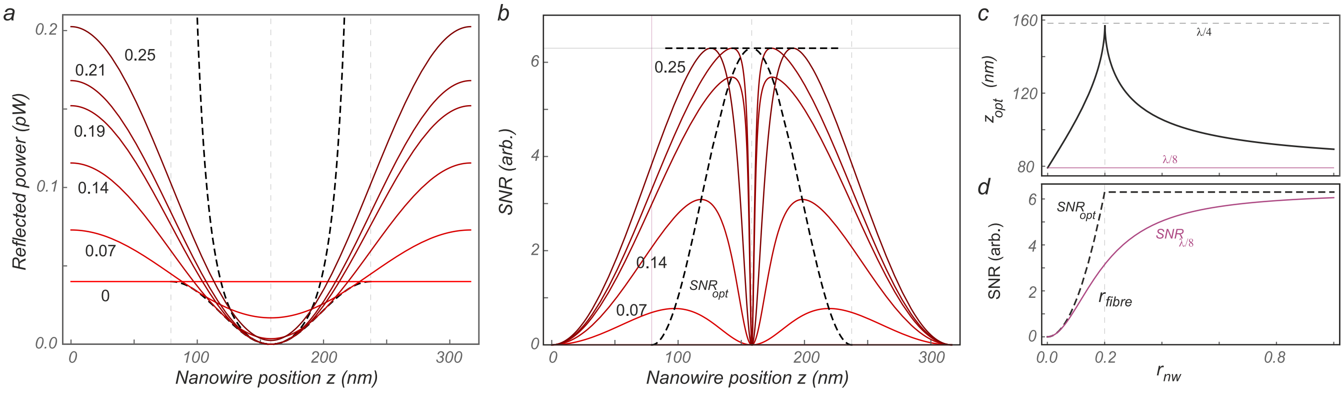

Supplementary Figure 6. SNR. a Reflected flux measured for a 1 pW injected power at 633 nm as a function of the nanowire position along the optical axis z, using $r_{\text{fiber}} = 0.2$ and for increasing values of $r_{\text{nw}} = \{0, 0.07, 0.14, 0.19, 0.21, 0.25\}$. **b** SNR expected as a function of the nanowire position, for the different nanowire reflectivities. The dashed lines are locii of maximum SNR for a given nanowire reflectivity. The optimum position and the optimum SNR achieved as a function of the nanowire reflectivity are reported in **c, d**. In case of a situation approaching the perfect dark fringe condition ($r_{\text{nw}} = r_{\text{fiber}}$), the SNR can be increased by a factor up to 2 compared to the SNR obtained at the maximum slope position by choosing the optimum position (z_{opt}).

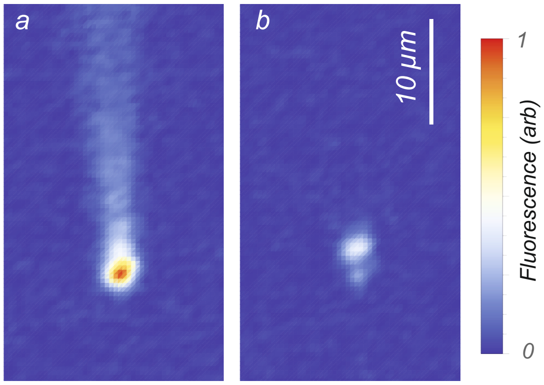

Supplementary Figure 7. Wave-guiding mechanism CCD fluorescence images obtained in reflection (600-750 nm wavelength span) of a nanowire illuminated by a focussed 532 nm laser spot (500 nm spot size) positioned at the nanowire extremity (a) and approx 2 μm above (b). The pump intensities and integration times are identical for both images. When the laser is positioned at the nanowire extremity, a fluorescence signal is observed along the nanowire, extending over tens of micrometers due to a wave-guiding mechanism. This is not the case, when the laser is positioned a few micrometers above the nanowire extremity. Note that the nanowire is tilted so that only its extremity is sharp on the image. We employed a fluorescence imaging technique in order not to be blinded by the reflected pump beam.

contrast increases, so does the SNR. The best shot noise sensitivity amounts to

$$S_{\delta r_\beta}^{\text{shotmin}} = \frac{\lambda^2}{64\pi^2 r_{\text{nw}}^2 \Phi_0}, \tag{32}$$

and the optimum position gets away from the position of maximum slope and gets closer to the minimum of reflected intensity (dark fringe). When the nanowire reflectivity is equal to or larger than r_{fiber}, the SNR is then maximized and the minimum shot noise level reaches

$$S_{\delta r_\beta}^{\text{shotmin}} = \frac{\lambda^2}{64\pi^2 r_{\text{fiber}}^2 \Phi_0}, \tag{33}$$

while the optimum readout position moves away from the dark fringe ($z = \lambda/4$) and converges again towards the side of the fringe. In such a situation it would be interesting to increase the input fiber reflectivity and potentially approaching a Fabry Perot regime. The optimum readout position is generally given by

$$2kz_{\text{opt}} = \begin{cases} \arccos(-r_{\text{nw}}/r_{\text{fiber}}), \text{if} & r_{\text{nw}} < r_{\text{fiber}} \\ \arccos(-r_{\text{fiber}}/r_{\text{nw}}), \text{if} & r_{\text{nw}} > r_{\text{fiber}} \end{cases}, \tag{34}$$

as shown in Supplementary Figure 6. We note that the dark noise of the photon counters, corresponding to a dark count rate of approx 50 cts/s (or 16 aW at 633 nm) is largely smaller than the shot noise level, so that it has not been taken into account here.

We can now come back to the abacus, and add the contour plot of P_0^{SNB} the injected power required to reach a SNB of 1 in the detection of the thermal noise peak, which verifies the equality $S_{\delta r_\beta}[\Omega_{\text{m}}] = S_{\delta r_\beta}^{\text{shotmin}}$. It is given by :

$$P_0^{\text{SNB}} \equiv h\nu \Phi_0^{\text{SNB}} = h\nu \frac{\lambda^2}{64\pi^2 r_{\text{nw}}^2} \frac{M_{\text{eff}} \Omega_{\text{m}}^3}{2k_B T Q}. \tag{35}$$

The dependance of the minimum readout power on the nanowire geometry has been added to the abacus of Supplementary Figure 3 as orange lines, where we have included an additional experimental losses (60 % quantum efficiency of the avalanche photodiodes, and 80 % collection efficiency of the reflected photon flux). The magnitudes obtained are in good agreement with our experimental observations, such as when a SNB of 1 was obtained on nanowire A with an injected readout power of 100 fW. To put those optical powers in perspective, one can remember that an input power of 1 pW generates in average a static heating of the nanowire up to approx 100 mK.

SUPPLEMENTARY NOTE 7 - ESTIMATION OF THE NANOWIRE CONDUCTANCE FROM THE OPTICAL HEATING CURVES

We first analyze the heating profiles created by a localized heat source (under the laser spot), and then present the extrapolation of the nanowire conductance from the measured optical heating curves. We adopt a diffusion-like model, but all the conclusions remains valid as long as one can define a proper temperature along the nanowire. This is not true anymore when the phonon mean free path becomes comparable to the nanowire length, the conduction then becomes ballistic in the nanowires, which occurs at temperatures lower than 200 mK (see next section).

Heating profiles and noise temperature

The equation governing the evolution of the temperature profile $T(y)$ along the nanowire can be written in absence of lateral loss:

$$\partial_t \left(\rho C T\right) = -\mathrm{div}\left(\mathbf{j}_{\mathrm{th}}\right) + p_{\mathrm{abs}}(\mathbf{r}) \tag{36}$$

where C is the local nanowire heat capacity that may depend on the temperature and thus indirectly in the position along the nanowire when the optical heating creates an appreciable temperature gradient. ρ is the density of SiC, that does not change with temperature. We have neglected here heat radiation which are inefficient at cryogenic temperatures (following the Stefan Boltzman law, the flux emitted by the nanowire surface is given by $\sigma T^4 2\pi R L$ which falls in the aW range already at 1 K). p_{abs} is the volumic heat source deposited in the nanowire, only located under the laser spot.

$$\mathbf{j}_{\mathrm{th}} \equiv -\kappa(T)\boldsymbol{\nabla} T \tag{37}$$

is the surfacic heat flux (in W/m^2) and κ the heat conductivity, that may also depend on temperature, and thus vary with the position along the nanowire.

We consider the static regime of the above equation to establish the static temperature profile in the nanowire and use a 1D model for simplicity. We assume that the tungsten tip is thermalized at T_0, the cryostat temperature, and that the heat is only deposited at the extremity of the nanowire, of length L, so that the boundary conditions can be written:

$$T(0) = T_0, \quad \text{and} \quad j_{\mathrm{th}}(L) = -\kappa(T(L)) \left.\frac{dT}{dy}\right|_L = -P_{\mathrm{abs}}/S, \tag{38}$$

$P_{\mathrm{abs}} = A_{\mathrm{abs}} P_0$ is the power absorbed, A_{abs} being the absorption coefficient, P_0 the injected power and S the section of the nanowire. Inside the nanowire, ($y = 0..L$) the differential equation becomes in the stationary regime:

$$\partial_y \left(\kappa(T(y))\partial_y T(y)\right) = 0 \tag{39}$$

We assume a temperature varying heat conductivity which can be written in the form of a power law:

$$\lambda(T) = \kappa_0 (T/T_0)^\mu \tag{40}$$

Solving the stationary differential equation, the stationary temperature profile can then be written as:

$$T(y) = T_0 \left(1 + (\mu+1) y \frac{P_{\mathrm{abs}}}{S\kappa_0 T_0}\right)^{1/(\mu+1)}. \tag{41}$$

If the heat conductivity does not depend on the temperature,$\mu = 0$ we have:

$$T(y) = T_0 + y\frac{P_{\mathrm{abs}}}{S\kappa_0} \tag{42}$$

which presents a linear increase along the wire and we obtain $T(L) - T_0 = \frac{L}{S\kappa_0} P_{\mathrm{abs}}$ where one can recognize the heat conductance $K^0 = \frac{S\kappa_0}{L}$ of the nanowire (in W/K). Using this definition, we can rewrite:

$$T(y) = T_0 \left(1 + (\mu+1)\frac{y}{L}\frac{P_{\mathrm{abs}}}{P_{\mathrm{warm}}}\right)^{1/(\mu+1)} \tag{43}$$

where we have introduced the threshold power:

$$P_{\text{warm}} \equiv T_0 K^0 \tag{44}$$

which is the absorbed power needed to double the temperature of the nanowire extremity (using the constant initial conductance). The temperature at the nanowire extremity is then:

$$T(L) = T_0 \left(1 + (1+\mu)\frac{P_{\text{abs}}}{P_{\text{warm}}}\right)^{1/(\mu+1)} \tag{45}$$

and for sufficiently large absorbed powers it can be approximated as:

$$T(L) \approx T_0 \left((1+\mu)\frac{LP_{\text{abs}}}{S\kappa_0 T_0}\right)^{1/(\mu+1)} \tag{46}$$

The noise spectral density of the total force driving a given eigenmode is given by an average of the local Langevin force noise spectral density, weighted by the normalized eigenmode profile $u_n(y)$, which verifies $\int_0^L u_n^2(y)dy = 1$:

$$S_{F_n}^{tot} = \int_0^L u_n^2(y) 2 m_{\text{lin}} \Gamma(y) T(y) dy, \tag{47}$$

where m_{lin} is the lineic mass of the nanowire and $\Gamma(y)$ the local damping rate which may depend on the temperature. When the damping rate does not depend on the temperature, as observed in our case (see manuscript), one can then connect the noise temperature T_n^{eff} of the mode to the temperature profile according to:

$$T_n^{\text{eff}} = \int_0^L u_n^2(y) T(y) dy. \tag{48}$$

When the heat conductance largely increase with temperature, the nanowire vibrating extremity, which is the warmest point in the nanowire presents a larger conductance than the clamping area. This means that the temperature profile in the nanowire will be more homogeneous and that the temperature gradients will be displaced towards the nanowire support where they weakly contribute to the noise temperature of the fundamental modes since they do not significantly vibrate there. In such conditions, it is then possible to assimilate their noise temperature to the temperature of the nanowire extremity: $T^{\text{eff}} \approx T(L)$. This remark simplifies the analysis of the heating curves used to extract the nanowire conductance dependence on temperature (see below). However one has to post-check that by computing the temperature profiles obtained using the determined conductance, they do not produce significant deviations once inserted in the above equation.

Derivation of the conductance

In absence of lateral losses, the heat flow within the nanowire is constant, and imposed by the absorbed optical power:

$$j_{\text{th}} = -P_{\text{abs}}/S = -\kappa(T(y))\,\frac{dT}{dy} \tag{49}$$

By integrating this equation along the nanowire, we obtain:

$$P_{\text{abs}} L/S = \int_0^L \kappa(T(y))\,\frac{dT}{dy} dy = \int_{T_0}^{T(L)} \kappa(T) dT, \tag{50}$$

where we have used the change of variable: $y \to T(y)$ (which is increasingly monotonous). Following the above arguments, in presence of a rapidly growing conductance with temperature, it is possible to assimilate the temperature of the nanowire extremity to the measured noise temperature. As such, the fitting expression we employ is:

$$P_0 = \int_{T_0}^{T^{\text{eff}}} \frac{K(T)}{A_{\text{abs}}} dT, \tag{51}$$

where we deduce the ratio $K(T)/A_{\rm abs}$ from the heating curve $(T^{\rm eff}, P_0)$ data set. The above expression is equivalent to:

$$\frac{K(T)}{A_{\rm abs}} = \frac{dP_0}{dT_{\rm eff}}, \tag{52}$$

which can be easier to exploit, but suffers more from imprecisions in the noise temperature evaluation compared to the integral form. This expression also summarizes the link between the temperature and power scalings of the conductance and noise temperature respectively ($K \propto T^{\mu} \Leftrightarrow T^{\rm eff} \propto P_0^{1/(1+\mu)}$). Its interpretation is also straight forward: when a certain equilibrium state is obtained, bringing the nanowire at given temperature, assumed homogeneous in the vibrating area, the achieved conductance simply connects incremental variations in noise temperature and absorbed optical power. We note that experimentally it could have been more adequate to vary the temperature of the cryostat to reach an homogeneous temperature profile within the nanowire, and for each temperature launch a heating curve: measuring the noise temperature for increasing optical powers, from which we can deduce the conductance. However this approach multiplies the required measurements, and takes an unbearable acquisition duration (acquiring a heating curves already took us a few days of averaging). Also, by using the mechanical properties of the nanowire, we indeed verified that our approach was valid: heating the nanowire with the cryostat or with the laser and using the measured noise temperature gives the same evolution, traducing that the hypothesis of a quasi-homogenous temperature profile inside the nanowire is indeed pertinent.

The heating curves realized with nanowires A and C are shown in Supplementary Figure 8. Several other nanowires were investigated, and the global trend is found to be robust. One may observe different heating efficiencies, even at different laser positions on the same nanowire, which reflect the possible variability of the absorption coefficient (in particular the heating efficiency is largely enhanced when placing the laser at less than 1-2 micrometers from the vibrating tip), but the presence of a "rapid" ($P_0^{1/2}$) increase below 100 mK, of a quasi-plateau in the 100-200 mK range, followed by a faster increase above 200 mK ($P_0^{1/2}$ then $P_0^{1/3}$) are robust common features. In the following, we will aim at understanding the presented heating curve which is the one realized with the largest optical span (almost 6 orders of magnitude). Measurement at high optical powers were realized by placing a calibrated optical density in front of the avalanche photodiode. The data are fitted using a $K(T)/A_{\rm abs}$ ratio reported in Supplementary Figure 8. We estimate the absorption coefficient by adjusting the nanowire conductance to the Casimir conductance at 10 K, see below, which determines the absorption coefficient at the level of 70 ppm. The rapid increase - over 4 orders of magnitude- of the deduced conductance over the temperature span, permits to validate the above employed hypothesis of a quasi-homogeneous temperature profile in the vibrating part of the nanowire. In the following section we will introduce the basics concepts that can help understanding the different regimes at play along those heating profiles.

SUPPLEMENTARY NOTE 8 - HEAT PROPAGATION IN THE NANOWIRE

The investigation of heat propagation in the nanowires represents a subject of research in itself, and will be the focus of future investigations, in particular in the ballistic regime. Here follow a few considerations on that topic, so that one can understand the different regimes at play in our experiment.
We only consider here heat propagation through phononic mechanisms and do not take into account possible electronic contributions due to a residual doping of the nanowires. For a complete interpretation of the measurements, one also has to consider the interfacial heat resistance at the nanowire-tungsten interface [6]. Rapid estimations from measurements realized on metal-dielectric materials (such as silicon - metal interfaces) suggest that this interfacial resistance should remain largely negligible compared to the extremely large thermal resistance of the nanowire. However the exact estimation of the interfacial resistance in our configuration is delicate.
The energy spectrum of the phonons in the material behaves as a black body distribution, which is peaked at the dominant phonon wavelength $\lambda_{\rm dom}$ defined as:

$$\lambda_{\rm dom} \equiv \frac{h v_s}{2.82 k_B T} \tag{53}$$

where the average speed of sound is $v_s = 13000\,{\rm m/s}$ in silicon carbide [7], and amounts to 0.7 nm, 70 nm and 7 μm at 300 K, 3 K and 30 mK respectively. At temperatures below 1 K, the dominant phonon wavelength becomes larger than the nanowire diameter, d, the nanowire progressively behaves as a 1D conductor, where the transverse phonon

modes do not participate anymore to the conduction. The second key parameter is the phonon mean free path $\Lambda_{\rm ph}$, which defines the coherence length of a phonon in the nanowire, the distance between two inelastic scattering events. At high temperatures, it is connected to the material conductivity $\kappa_{\rm th}$ and heat capacity $C_{\rm th}$ by the kinetic equation: $\kappa_{\rm th} = \Lambda_{\rm ph} v_s C_{\rm ph}/3$. At room temperature the mean free path is smaller than the nanowire diameter, that that its heat conduction properties can be deduced using the expressions valid for bulk material. When lowering the temperature, the mean free path progressively increases and becomes comparable to the nanowire diameter. It can even extend up to a few mm at helium temperatures in the bulk. However, in the case of a nanowire, the phonon undergoes "reflections" not only on material defects but also on the nanowire surface, which can be either specular or diffusive. The type of reflection observed depends how the wavelength (and orientation) of the phonon mode considered compares to the rugosity of the nanowire surface. If the nanowire rugosity η is larger than the phonon wavelength, the phonon looses its spatial coherence upon reflection and the mean free path becomes limited to the nanowire diameter. This is called the Casimir regime [8]. At lower temperatures, the dominant phonon wavelength further increases, and progressively becomes larger than the nanowire rugosity. By doing so, the phonons dominantly contributing to the heat conduction will undergo multiple reflections without losing their spatial coherence, so that the average mean free path progressively increases beyond the nanowire diameter. This progressive increase of the mean free path at low temperatures is called the Ziman regime.

A typical nanowire rugosity estimation falls in the 1-10 nm range, dominated by the contribution of the oxyde crust. Larger diameter modulations can appear in presence of allotropic defects (presence of small segments, 100-500 nm in length, of a different allotropic phase), but the nanowire employed in this work were selected to avoid such diameter changes. For larger rugosities, it is necessary to reach a larger dominant phonon wavelength to see an increase of the mean free path beyond the nanowire diameter, and the deviation arises at lower temperatures.

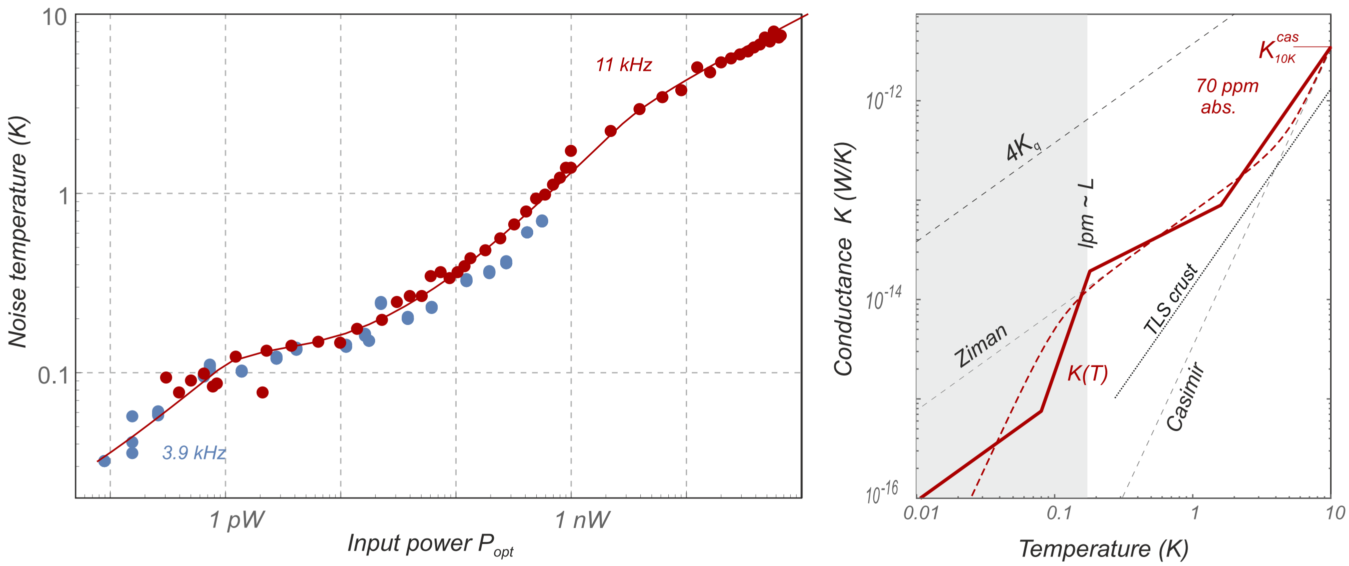

Supplementary Figure 8. **Conductance Analysis** Left: optical heating curve realized on nanowire C on a larger power span. The measurements obtained with nanowire A are also shown in blue and follow the same trend, in particular the plateau region observed in the 100-150 mK range of effective noise temperature. The solid line is a fit to the data using a temperature dependent conductance, which is reported in the right panel. Those measurements do not allow to separately determine the absorption coefficient and absolute nanowire conductance, but the latter can be estimated at 10 K using the Casimir model (see text). In that case, the nanowire absorption coefficient obtained is of 70 ppm. The dashed line represents the ballistic quantum conductance for comparison. The Casimir and Ziman regimes are shown, using a $\eta = 3$ nm rugosity parameter for the latter, which permits to estimate the average phonon mean free path becomes comparable to the nanowire length below temperatures of 200 mK, when the nanowire enters the plateau area, which corresponds to a very rapide increase of the nanowire conductance. The linear increase observed below 100 mK is likely to be the regime where only the fundamental transverse mode of phonons in the nanowire participates to the heat conduction, with a very poor transmissivity (less than 1 percent), which can be explained by the inadequate contact geometry. The dotted black line represents the conductance of the oxyde crust, assuming it behaves as an amorphous ring of 3 nm thickness around the nanowire [5].

In the Casimir regime, the nanowire conductance is given by [8]:

$$K(T) = 3200 \left(\frac{2\pi^2 k_B^4}{5\hbar^3 v_s^3}\right)^{\frac{2}{3}} \frac{\pi(d/2)^2\Lambda_{\rm ph}}{L} T^3, \tag{54}$$

where the mean free path $\Lambda_{\rm ph}$ is assimilated to the nanowire diameter. This estimation gives a conductance of $K_{\rm cas} = 3.5\,{\rm pW/K}$ at 10K for our $245\,\mu{\rm m}, 130\,{\rm nm}$ nanowire (sample C), see Supplementary Figure 8. As already observed experimentally in many experiments, this value is significantly smaller than the conductance estimation based on the bulk conductivity measured at a level of $\kappa_{\rm sic} = 30\,{\rm W/m/K}$ at 10 K, which gives $K_{\rm bulk} = \kappa_{\rm sic}\pi(d/2)^2/L = 1.6 \times 10^{-9}\,{\rm W/K}$. With the above evaluation of the Casimir conductance at 10 K, we obtain an optical absorption coefficient of 70 ppm.

At smaller temperatures, the dominant phonon wavelength increases, and at some point one can observe a transition from the Casimir to the Ziman regime, when the phonon reflections on the nanowire periphery progressively become elastic/specular. This transition depends on the rugosity profile (rms amplitude η) of the nanowire, which generates specular/diffusive reflections for phonon wavelength larger/smaller than η. A correction to the Casimir model can then be introduced, by replacing the Casimir mean free path $\Lambda_{\rm ph} = d$ by the Ziman correction:

$$\Lambda_{\rm Ziman} = d\,\frac{1 - p_{\rm spec}}{1 + p_{\rm spec}} \tag{55}$$

where $p_{\rm spec}$ represents the probability of specular reflection, evaluated at the dominant phonon wavelength, and can be expressed as: $p_{\rm spec} = \exp\left(-16\pi^3\eta^2/\lambda^2_{\rm dom}(T)\right)$. For our nanowires, the average rugosity parameter is around $\eta = 3\,{\rm nm}$. As a consequence, one can observe a slower decrease of the conductance with decreasing temperature ($\propto T$ vs $\propto T^3$), traducing that the heat conduction is more efficient in presence of specular reflections.

At lower temperature, the Ziman mean free path $\Lambda_{\rm Ziman}$ of the phonons oscillating at the dominant phonon wavelength becomes comparable to the nanowire length L, which represents an upper limit for the model validity. For our nanowires, it is reached at a temperature around 150-200 mK. Below this temperature, the nanowire conductance gets sharply reduced and the nanowire enters the ballistic regime. In the optical heating curves, this generates a quasi flat behavior, which ends around 150-200 mK and has been systematically observed on the different nanowires employed. In Supplementary Figure 8, we employ the following expression of the mean free path:

$$\Lambda^{-1} = L^{-1} + \Lambda^{-1}_{\rm Ziman} \tag{56}$$

in the Casimir formula. The curve is plotted as a red dashed line, and presents a qualitative agreement with the experimentally deduced nanowire conductance (full red line), in the high temperature regime, above 200 mK, despite the simplicity of the model employed.

The sharp slope (T^5 or higher) observed in the conductance is then due to the fact that progressively all the phonon wavelengthes contributing to the conduction mechanism see their mean free path topped to the nanowire length. Finally the linear T dependence of the conduction observed at temperature lower than 100 mK should correspond to the quantum conductance regime, which amounts for the 4 polarizations of the fundamental transverse mode to $4K_Q(T) = 4\pi^2 k_B^2 T/3h$ in case of perfect transmission at the interfaces. In that regime, only the longitudinal mode contribute, and it becomes visible once the transverse modes do not participate anymore in the conduction mechanism. This happens when $\lambda_{\rm dom} > d$, which happens around a few hundreds of mK. The expected level of the ideal quantum conductance is shown in Supplementary Figure 8, and falls well above our estimated nanowire conductance, meaning that our transmission factor would be around 0.3 %. This rather low value is not really surprising since no impedance matching geometry is used here since it is incompatible with the large decoupling from the nanowire and its support required to operate with large mechanical quality factors.

The contribution from the oxyde crust can be estimated using the conductance derived from the kinetic equation for bulk amorphous solids [5]: $\kappa(T) \approx \rho k_B^3/(6\pi\hbar^2)3v_s/(\bar{P}\gamma^2)T^2$, where the constant $\bar{P}\gamma^2/\rho v_s^2 \approx 10^{-4}$ is universal for amorphous materials. The contribution of an oxyde crust 3 nm in thickness extended all along the nanowire is shown for comparison in Supplementary Figure 8. Its value is smaller but comparable to the Casimir conductance at high temperatures, but becomes negligible at lower temperatures. However, the presence of the oxyde is likely to have a important impact on the phonon scattering properties on the nanowire boundaries, and thus to affect the Ziman-Casimir transition.

We insist that the above considerations are only quantitative. They permit to identify the different conduction regimes at play in our system, evolving from a purely ballistic 1D regime to the Casimir regime. A more refined

analysis, taking into account the different phonon modes and the individual evolution of their mean free path [9–11] should help reaching a quantitative description of the heat conduction regimes in our system. This will the the topic of future investigations and falls beyond the scope of this paper.

SUPPLEMENTARY NOTE 9 - FORCE SENSING ON THE 11 KHZ NANOWIRE

Supplementary Figure 9 presents some additional data that complement the measurements introduced in Fig.5 of the article.

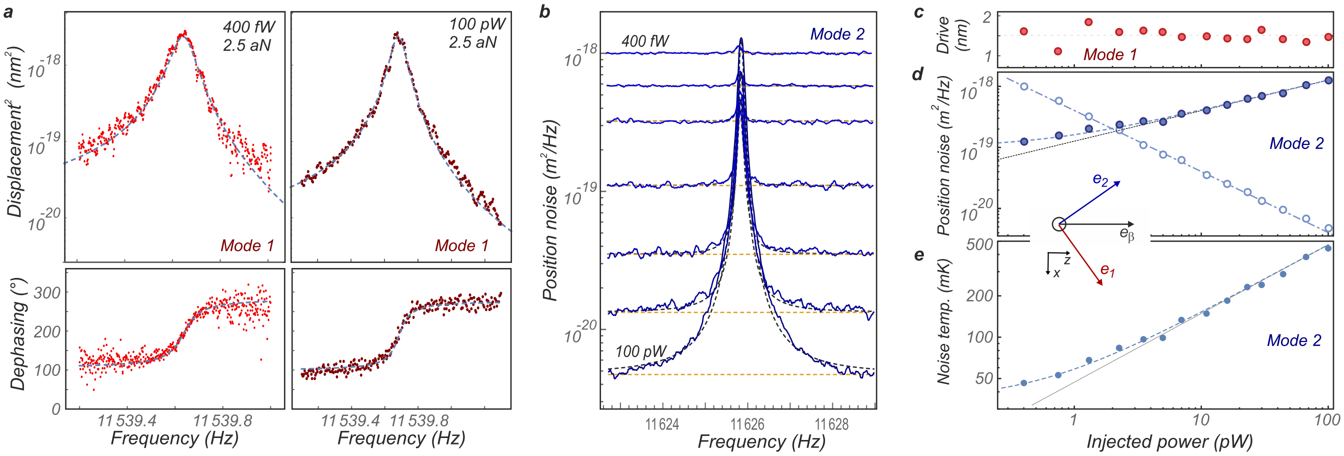

Supplementary Figure 9. **Nanowire 11kHz.** Additional information to Fig. 5cde. One of the nanowire fundamental eigenmode is piezo-driven with a rather intense force of 2.5 aN in amplitude, while its thermal noise is measured on the second eigenmode which serves as a thermometer. The mode orientations ($\mathbf{e_1}, \mathbf{e_2}$) are rotated by 36 with respect to the (x,z) basis. The measurement vector $\mathbf{e}_\beta$ is aligned with the optical axis (z), see sketch. **a** Driven response measured with a lock-in (amplitude and phase) while sweeping the drive tone across the low frequency mode (mode 1) for the 2 extreme optical readout powers employed: 400 fW and 100 pW. The resonant driven amplitude is reported in panel c. The dephasing induced by the piezo actuation chain has not been subtracted. **b** Thermal noise spectra measured on mode 2, for increasing injected optical powers $(0.4, 0.75, 1.3, 3.5, 11, 30, 100\,\mathrm{pW})$. The amplitude of the fitted thermal noise peak and the background level are reported in panel d, while the noise temperature is reported in panel e. **c** Driven oscillation amplitude (projected along $\mathbf{e}_\beta$) for increasing injected optical power. **d** Resonant noise spectral density $S_{\delta r_\beta}[\Omega_2]$ (full circles) and background level (open circles) measured for increasing injected optical powers. As expected for a shot noise limited detection, the background level scales inversely with the injected power (dot-dashed line). The full line represents a $P^{0.5}$ power law. **e** Corresponding noise temperatures measured on mode 2 for increasing optical powers while continuously driving the first eigenmode. The full line is a $P^{0.5}$ power law, similar to the one observed on nanowire A (see manuscript) at minimal powers. The minimal noise temperature is of 47 mK, without the suspension apparatus, which confirms that the parasitic vibration noise level due to the mixture circulation strongly depends on frequency.

SUPPLEMENTARY NOTE 10 - FREQUENCY STABILITY OF THE NANOWIRES

Supplementary Figure 10 illustrates measurements of the frequency stability of nanowire A at cryogenic temperature. When measurements of force field gradients are realized at a measurement pace of one measure every $2\pi/\Gamma_\mathrm{m}$, sufficient to let the nanowire reach its stationary state, the measured relative frequency stability of $\Delta\Omega_\mathrm{m}^\tau/\Omega_\mathrm{m} = 5\times10^{-11}$ leads to a minimum detectable force gradient of $\nabla F^\mathrm{min} = 2\Omega_\mathrm{m}^2 M_\mathrm{eff}\cdot\Delta\Omega_\mathrm{m}^\tau/\Omega_\mathrm{m} = 0.9\,\mathrm{fN/m}$. Similar measurements realized with a larger nanowire (nanowire D, 800 μm length, 500 nm diameter) oscillating at 1507-1513 Hz with a damping rate of $\Gamma_\mathrm{m}/2\pi = 8\,\mathrm{mHz}$ and an effective mass of 200 pg, lead to a smaller frequency deviation approaching $40-48\,\mu$Hz over 4 hours, and a relative Allan deviation of 4×10^{-11} for a gate time of $\tau = 125s$ for both modes. This leads to similar lateral force gradient sensitivities (0.7 fN/m).

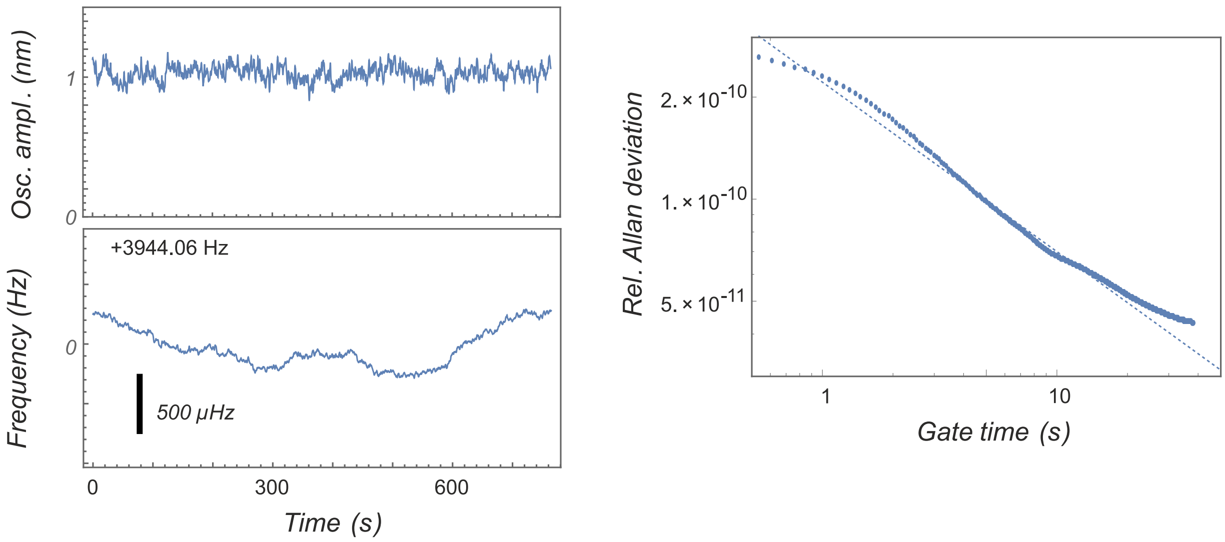

Supplementary Figure 10. **Frequency stability** Left: typical measurements of the frequency stability of NW A, realized at 27 mK. The nanowire is resonantly driven up to an oscillation amplitude of approx. 1 nm using a piezo actuation and the displacement signal is demodulated at the driving tone frequency (200 mHz bandwidth, data transfer rate of 13 Hz here). A deviation of 160 μHz is measured over approx. 10 minutes, after substraction of a linear drift. Right: computed relative Allan deviation obtained after subtraction of the linear drift. The dashed line is a $\tau^{-1/2}$ guide line.

SUPPLEMENTARY NOTE 11 - CONVERGENCE OF THE FIT FUNCTION

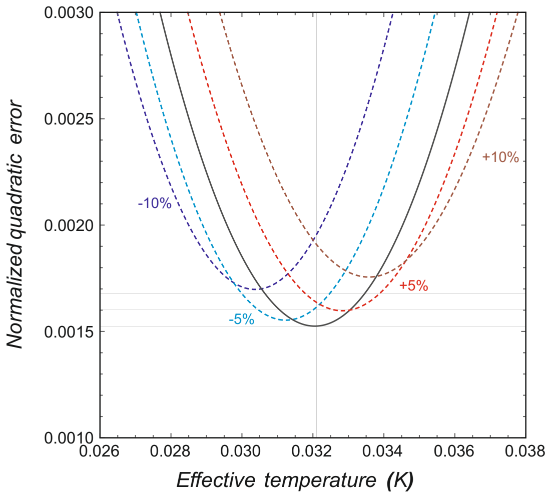

Supplementary Figure 11. **Convergence plot of the fit function** Plot of the normalized quadratic error of the thermal noise fits (for the coldest measurement shown in Fig. 3), as a function of the effective temperature for different values of the damping rate $(-10, -5, 0, +5, +10\%$ change imposed around to the best value (53.8 mHz). The smallest error value for the different traces are obtained for 30.4, 31.2, 32.06, 32.8, 33.5 mK respectively. The horizontal lines represent a 0, +5 and +10 % change with respect to the minimum normalized quadratic error ($\approx 0.15\%$). The shot noise level, which can be averaged on many frequency points far from resonance, is identical for all curves.

The convergence of the fitting function for the coolest measurement shown in Fig 3. is reproduced in Supplementary Figure 11. The normalized quadratic error of the fits, defined as $\sum_i (S_{\delta r_\beta}[\Omega_i]^{\rm data} - S_{\delta r_\beta}[\Omega_i]^{\rm fit})^2 / \sum_i (S_{\delta r_\beta}[\Omega_i]^{\rm data})^2$ is calculated for different effective temperatures, while imposing different damping rates varying on $\pm 10\%$ around the best fit value. A $\pm 5\%$ variation in the fitted damping rates leads to a $\approx \pm 2.5\%$ change in the effective temperature.

Experimentally we observe a statistical variation of ±5% on those two parameters while fitting several thermal noise spectra taken in identical measurement conditions.

SUPPLEMENTARY REFERENCES

* corresponding author: olivier.arcizet@neel.cnrs.fr

[1] Bohren, C. F. & Huffman, D. *Absorption and Scattering of Light by Small Particles* (WileyVCH, Berlin, 1983).

[2] Bechelany, M., Brioude, A., Cornu, D., Ferro, G. & Miele, P. A Raman spectroscopy study of individual SiC nanowires. *Adv. Funct. Mater.* **17**, 939 (2007).

[3] Mercier de Lépinay, L. *Habillage mécanique d'un nanofil par un champ de force : de la mesure vectorielle ultrasensible aux systèmes quantiques hybrides.* Ph.D. thesis, Université Grenoble Alpes (2017).

[4] Iglesias, I. & Sáenz, J. J. Scattering forces in the focal volume of high numerical aperture microscope objectives. *Opt. Commun.* **284**, 2430 (2011).

[5] Enss, C. & Hunklinger, S. *Low-Temperature Physics* (Springer-Verlag Berlin Heidelberg, 2005).

[6] Swartz, E. T. & Pohl, R. O. Thermal boundary resistance. *Rev. Mod. Phys.* **61**, 605 (1989).

[7] Harry, G. L. (ed.) *Properties of Silicon Carbide* (INSPEC, 1985).

[8] Casimir, H. B. G. Note on the conduction of heat in crystals. *Physica* **5**, 495 (1938).

[9] Callaway, J. Model for lattice thermal conductivity at low temperatures. *Phys. Rev.* **113**, 1046 (1959).

[10] Holland, M. G. Analysis of lattice thermal conductivity. *Phys. Rev.* **132**, 2461 (1963).

[11] Anghel, D. V. & Manninen, M. Behavior of the phonon gas in restricted geometries at low temperatures. *Phys. Rev. B* **59**, 9854 (1999).
